# Dendritic Cell-Based Immunotherapy Treatment for Glioblastoma Multiforme

**DOI:** 10.1155/2015/717530

**Published:** 2015-06-17

**Authors:** Liu Yang, Geng Guo, Xiao-yuan Niu, Jing Liu

**Affiliations:** ^1^Department of Neurology, The First Hospital, Shanxi Medical University, No. 85 South Jie Fang Road, Taiyuan, Shanxi 030001, China; ^2^Department of Neurosurgery, The First Hospital, Shanxi Medical University, No. 85 South Jie Fang Road, Taiyuan, Shanxi 030001, China

## Abstract

Glioblastoma multiforme (GBM) is the most malignant glioma and patients diagnosed with this disease had poor outcomes even treated with the combination of conventional treatment (surgery, chemotherapy, and radiation). Dendritic cells (DCs) are the most powerful antigen presenting cells and DC-based vaccination has the potential to target and eliminate GBM cells and enhance the responses of these cells to the existing therapies with minimal damage to the healthy tissues around them. It can enhance recognition of GBM cells by the patients' immune system and activate vast, potent, and long-lasting immune reactions to eliminate them. Therefore, this therapy can prolong the survival of GBM patients and has wide and bright future in the treatment of GBM. Also, the efficacy of this therapy can be strengthened in several ways at some degree: the manipulation of immune regulatory components or costimulatory molecules on DCs; the appropriate choices of antigens for loading to enhance the effectiveness of the therapy; regulation of positive regulators or negative regulators in GBM microenvironment.

## 1. Introduction

Glioblastoma multiforme (GBM) is the most malignant glioma, accounting for 60–70% of all gliomas [1]; 88% of all GBM patients die within 3 years [2]. Complete surgical resection is difficult to perform due to tumor infiltration into the brain parenchyma and eventual tumor relapse [3]. The median survival time (MS) is 12–15 months with conventional treatment (surgery, chemotherapy, and radiation) for primary GBM patients [1], and 3–6 months for recurrent GBM patients [4].

Therefore, new treatment options are needed to improve patient outcomes for this unmet clinical need. Immunotherapy may be a successful treatment option with the advantage of high tumor-specific targeting [5]. Several reagents have recently gained the Food and Drug Administration (FDA) approval and have demonstrated clinical benefit [6, 7]. The focus of immunotherapy vaccines is based upon the concept that antigen presenting cells (APCs) can effectively be loaded with tumor derived antigens that will accelerate tumor eradication within* in vivo* settings [8]. Dendritic cells (DCs) are the most powerful human APCs and DC-based vaccines have the potential to improve clinical outcomes by enhancing GBM cell responses to existing therapy and/or stimulating innate immune responses with minimal toxicity. Ultimately, vaccination should enhance recognition of GBM cells by the patients' immune system and increase activity of tumor-infiltrating lymphocytes (TILs) against them [9], creating potent, long-lasting tumor-specific T lymphocytes. Within the context of this paper, we review DC-based vaccination for GBM patients as shown in Algorithm 1.

## 2. Dendritic Cells (DCs)

DCs are at minimum, large, granular lymphocytes with high cell surface markers: major histocompatibility complex (MHC) class I molecules, MHC class II molecules, and CD86, all of which can help identify DCs from other myeloid lineage cells [10]. They recognize and capture antigens in their immature state and then migrate to lymphoid organs where they present processed peptides (derived from captured antigens) to T cells in the context of MHC I or II [11, 12] and therefore induce tumor antigen-specific immune responses. They also display various characteristics in immune regulatory systems that balance the complex system of inflammatory and inhibitory immune reactions in the tumor microenvironment [3]. Therefore, they are involved in aspects of both innate and adaptive immune systems and can modulate immune functions, reverse immune suppression, and decrease tumor immune tolerance and therefore terminate low immunoreactivity in tumor patients [13].

### 2.1. Selection of DC Subpopulations

**Figure 1:**
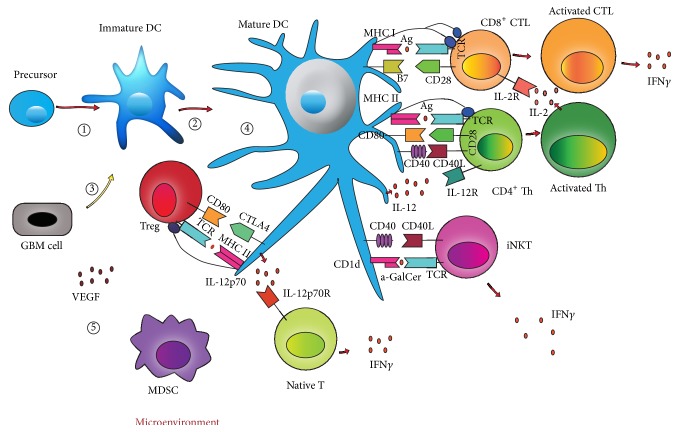
Dendritic cell (DC)-based vaccination immunotherapeutic strategies for glioblastoma multiforme (GBM). Bone-marrow derived precursors are differentiated into DCs by Flt3L or GM-CSF. DCs can be divided into two distinct subtypes, types 1 and 2. They act differently and have synergistic effects in antitumor immunity. They can be loaded with GBM antigens derived from RNA, DNA, proteins, peptides, lysates, glioma stem cells antigens, apoptotic cells or fusion. They recognize and capture antigens, then they present processed peptides (derived from captured antigens) to T cells in the context of major histocompatibility complex (MHC) class I or II (signal 1). Then pulse tumor-loaded DCs with maturation stimuli to increase the expression of costimulatory molecules such as CD80 (signal 2) and the secretion of proinflammatory cytokines such as IL-12 (signal 3). Then CD4+ helper T cells secrete IL-2 to stimulate CD8+ cytotoxic T cells which then secrete IFN-*γ* and exhibit cytolytic immune responses against GBM cells. Upregulating costimulatory signals or suppressing coinhibitory signals can strengthen the efficacy of DC vaccines. Manipulation of these signals includes: TLR agonists, CD40 ligand, CD70, tumor necrosis factor receptor superfamily-member 4 (TNFRSF4) ligandDi, iNKTs agonists, and silencing A20 or SOCS1 by siRNA et al. Moreover, regulation of GBM microenvironment also can enhance the efficacy of DC vaccines. These regulation includes: the addition of some leukocytes and cytokines, Treg depletion, MDSCs inhibition, and VEGF inhibition et al. Ag: antigen, CTL: cytotoxic T-cell, CTLA-4: cytotoxic T-lymphocyte antigen 4, DC: dendtiric cell, DC1: type 1 polarizing DC, DC2: type 2 polarizing DC, Flt3L: fms-like tyrosine kinase 3 ligand, GM-CSF: glanulocyte monocyte-colony stimulating factor, IFN: interferon, IL: interleukin, iNKTs: Invariant natural killer T cells, MDSC: myeloid-derived suppressor cell, MHC: major histocompatibility class, siRNA: small interfering RNA, SOCS1: suppressor of cytokine signaling 1, TCR: T cell receptor, Th: helper T cells, TLR: Toll-like receptor, Treg: regulatory T cell, VEGF: vascular endothelial growth factor. ① Differentiation: GM-CSF/Flt3L. ② Selection of subpopulation: DC1/DC2. ③ Antigen loading: RNA, DNA, proteins, peptides, lysates, glioma stem cell antigens, fusion, and apoptotic cells. ④ Manipulation signals in DCs: TLR agonists, CD40 ligand, CD70, TNFRSF4 ligandDi, iNKTs agonists, silencing A20 or SOCS1 by siRNA. ⑤ Regulation of GBM microenvironment: the manipulation of some leukocytes and cytokines, Treg depletion, MDSCs inhibition, and VEGF inhibition.

DCs can be divided into two distinct subtypes, types 1 and 2. Type 1 polarizing DC (DC1) subsets are associated with antitumor immunity as they direct effector T cell responses to the helper T cell 1 (Th1) phenotype, whereas the DC2 subset is vital for antitumor immunity against extracellular antigens (Figure 1). DC1 polarization induces abundant production of interleukin (IL)-12p70 heterodimer and IL-23, secretion of chemokine MIP-1, and expression of Delta-4 Notch ligand [14]. Products induced by DC1 are associated with chemoattraction and activation of Th1-type CD4^+^ and CD8^+^ T cells. Moreover, IL-12p70 is critical for the sensitization of high-avidity T cells which recognize and kill tumor targets directly [3, 14–16]. Therefore, the choice of DC1 may be inviting.

### 2.2. DC Differentiation

DC differentiation from bone marrow (BM) precursors can be induced by granulocyte macrophage colony-stimulating factor (GM-CSF) or FMS-like tyrosine kinase-3 ligand (Flt3L) (Figure 1). GM-CSF expands both DC1 and DC2 subsets, yielding more DC2 than DC1 cells, whereas Flt3L preferentially expands the DC1 subset. Both Flt3L and GM-CSF increase naive and memory T cells in mice, but memory CD4^+^ and CD8^+^ T cells are increased more by Flt3L compared to GM-CSF. GM-CSF increases the frequency of both Th1 and helper T cell 2 (Th2) cells, and Flt3L mainly increases Th1 cell frequency. DC1 isolated from Flt3L-injected mice had more IL-12p40 than IL-10, compared to DC2 [17, 18] (Table 1).

When BM cells were cultured with GM-CSF, followed by interferon (IFN)-*γ*, IFN-*α*, IL-4 and polyinosinic-polycytidylic acid (polyI:C), the proportion and function of the DC1 subset in GM-CSF-treated progenitor cells were increased. Such *α*-type-1 polarized DCs produced more IL-12 compared to the normal DC1 subset and they were more resistant to the immunosuppressive environment created by regulatory T cells (Tregs) [16]. Also *α*-type-1 polarized DC vaccines loaded with GBM antigens could effectively control GBM relapse by inducing Th1 and cytotoxic lymphocyte (CTL) responses and suppressing accumulation of Tregs in Draining Lymph Nodes (DLNs) in mouse models [15]. When mouse BM cells are cultured with Flt3L, followed by IL-6 stimulation, CD34^+^ progenitor cells are expanded and then differentiated into DCs [19].

DCs conditioned from GM-CSF and DCs conditioned from Flt3L have different properties, and cell population admixtures may be best for DC preparations [3]. When Flt3L and GM-CSF were combined, DC infiltration into mouse tumors was inhibited and Tregs were activated, thereby promoted tumor tolerance [19]. And the combined cytokine regimen seems to increase the number of tumor-infiltrating dendritic cells (TIDCs) that can induce antigen-specific CD8^+^ T cells but also CD4^+^ Tregs that may neutralize the antitumor activity of the CD8^+^ T cells in situ [20]. But the method culturing DCs from humans by using GM-CSF and Flt3L remains to be explored.

### 2.3. Manipulation of Costimulatory and Coinhibitory Signals via DCs

Many costimulatory and coinhibitory molecules found on DCs function differently in varying immune response situations. Upregulating costimulatory signals or suppressing coinhibitory signals can strengthen the efficacy of DC vaccines (Figure 1).

The presence of immunosuppressive conditioning (such as IL-10 or IL-27) or costimulatory molecule expression insufficiency (B7-1) or proinflammatory cytokine secretion (IL-12) can induce tolerogenic DCs, which express coinhibitory molecules and secrete immunosuppressive cytokines, subsequently inducing tolerance. Tolerogenic DCs can secret soluble factors which attract Tregs to the tumor microenvironment. These factors include chemokines CCL17 and CCL12, which bind to CCR4 and CCR8 receptors on Tregs [21]. Blockade of CCL17 and CCL22 can reduce Tregs migration to the tumor microenvironment, sustaining sufficient antitumor immunity [3].

#### 2.3.1. Positive Regulators

Costimulatory molecules are important for the induction of immune responses. Robust T cell responses not only need a signal induced through T cell receptors via recognition of antigenic peptide MHC molecules on DCs, but also call for signals provided by interactions of costimulatory ligands on T cells and their receptors on DCs [3, 22, 23]. Antigen-specific T cells become anergic in the absence of costimulatory molecule interactions [24]. Therefore, the therapeutic immunity of DC vaccines can be strengthened by upregulating the costimulatory molecules [25].

Costimulatory molecules belong to two major families: the B7/CD28 family and the tumor necrosis factor (TNF)/TNF receptor family. B7/CD28 family members are involved in initiation of cell-mediated immune responses, while TNF/TNF receptor family members are involved in the later phases of T-cell activation. B7 molecules expressed on DCs include CD80 (B7-1), CD86 (B7-2) [25], inducible costimulator (ICOS) ligand (B7-H2) [26], programmed death 1 ligand (PD-L1 or B7-H1), PD-L2 (B7-DC), B7-H3 [27], and B7-H4 [28]. TNF/TNF receptors include CD27, 4-1BB (CD137), tumor necrosis factor receptor superfamily-member 4 (TNFRSF4), tumor necrosis factor ligand superfamily-member 14 (TNFSF14), and glucocorticoid-induced tumor necrosis factor receptor (GITR) [29–31]. B7-1 and B7-2 bind two surface molecules on T cells, the stimulatory receptor CD28 and the inhibitory receptor CTLA-4 (CD152). The engagement of CTLA-4 by B7-1 or B7-2 downregulates immune responses thereby leading to immune tolerance and profound autoimmunity driven by self-reactive T cells that are converse to the engagement of CD28 which promotes T cell activation [27]. Therefore, to strengthen the antitumor immune responses, blockade of signaling transduced through CTLA-4 is essential in addition to upregulation of B7-1 and B7-2 by immunostimulants [32].

Expression of costimulatory molecules in DC vaccines can be increased by the pulse of some agents for maturation [3]. These agents include Toll-like receptor (TLR) agonists, CD40 ligand, CD70, TNFRSF4 ligand, calcium ionophores, and GITR ligand [3, 14, 33]. TLR agonists include follistatin-like 1 (FSL-1) and macrophage-activating lipopeptide 2 KDa (MALP2; TLR2/6 agonist), Pam3Cys (TLR1/2 agonist), polyI:C (TLR3 agonist), lipopolysaccharides (LPS) and monophosphoryl lipid A (MPL-A; TLR4 agonists), imiquimod and class B CpG oligodeoxynucleotide (CpG; TLR9 agonist), and R848 (TLR7 agonists) which inconsistently stimulate immune responses. For example, TLR1/2 and TLR3 agonists can induce responses from DC1, while TLR3/4 + TLR7/9 agonists mainly induce responses from DC2 [34]. Utilization of TLR agonists could enhance survival and trafficking of DCs* in situ* as well as prime tumor antigen-specific T lymphocytes [35]. TLRs are widely expressed in immune cells and tumor cells in which expressed preferentially [36] and the expression of TLRs on immune or GBM cells in the GBM microenvironment affects the therapeutic effect of TLR agonists. GL261 cells express TLR2, TLR3, and TLR4 and can increase MHC I expression and induce IL-6 secretion in respond to the corresponding TLR ligands [36]. When DCs are activated by TLR signals, they upregulate costimulatory molecules; secrete immunomodulatory cytokines (IL-12), and increase antigen procession and presentation to B and T lymphocytes. Intratumoral injection of TLR1/2 or TLR7 agonists produced a survival benefit, and TLR9 agonists had the best therapeutic effect for GL261 glioma cells, compared to less effective stimulation by TLR3 and TLR4 agonists alone [36]. Combining synergistic immunostimulants can elevate immune responses against GBM. For example, combining CD40 and TLR ligands significantly suppresses tumor growth in mice with melanoma [37].

Invariant natural killer T cells (iNKTs) are a subset of T cells that recognize glycolipid antigens bound to CD1d (a MHC Class I-like molecule highly expressed on DCs) by semi-invariant *αβ* T-cell receptors [38]. They license DCs to initiate adaptive immune responses via CD40-CD40 ligand interactions between DCs and iNKTs. The potent synthetic iNKTs agonist *α*-galactosylceramide (*α*-GalCer) can promote T-cell responses to DC vaccines. DCs acquire*α*-GalCer and present it to CD1d molecules, and then DCs rapidly express immunostimulatory factors such as CD40 upon interaction with iNKTs, inducing enhanced capacity to drive conventional T-cell responses [39] (Figure 1).

#### 2.3.2. Negative Regulators

Many negative regulators suppress immune responses and the blockage of these molecules may offer promise for increasing therapeutic efficacy of DC-based vaccination. PD-L1, PD-L2, and B7-H4 are costimulatory molecules which downregulate T-cell immune responses [28, 40]. Many GBM patients have aberrant expression of PD-L1 which correlates with a poor prognosis [22]. Blockade of PD-L1 and PD-L2 on DCs by an antibody can improve proliferation and cytokine production of CD4^+^ T cells [41].

DCs also express molecules that may suppress antigen presentation or activation and function of T cells. The knockdown expression of these molecules by small interfering RNA (siRNA) can increase antitumor immunity mediated by DCs. These suppressive molecules include: zinc finger protein A20 (A20; a negative regulator of TLR and the TNF receptor signal pathway which stimulates T-cell mediated responses) [42], and the suppressor of cytokine signaling 1 (SOCS1; a negative regulator signaling through IFN-Γ, IL-2, IL-6, or IL-12, stimulators in T-cell expansion) [43]. Silencing A20 or SOCS1 in antigen-loaded DCs by siRNA caused DCs to activate a large amount of effector T cells and this was correlated to tumor growth inhibition in mice [3].

DC-derived immunoglobulin receptor 2 (DIgR2) and Notch ligands are surface molecules which direct suppressive effects on T cells and they are targets for increasing therapeutic efficacy of DC vaccines. When Delta1, a Notch ligand, is silenced by siRNA, cytokines secreted by CD4^+^ T cells were increased in response to polyclonal T cell receptor activation [44] (Table 2).

## 3. Loading Antigens

The effectiveness of uptaking and loading GBM antigens on MHC complexes of DCs and expansion of DC subgroups which prime naïve T cells affect the therapeutic efficacy of DC vaccines. Thus, it is important to choose appropriate antigens for loading (Figure 1).

### 3.1. Glioblastoma-Associated Antigens (GAAs) and Glioblastoma Specific Antigens (GSAs)

GBM antigens include GAAs and GSAs. Antigens loaded on DCs include RNA, DNA, proteins, peptides and lysates, or fusion and apoptotic cells [45]. Many identified GAAs been used for DC vaccination—antigen isolated from immunoselected melanoma-2 (AIM-2) [46], the *α*-2 chain of the IL-13 receptor (IL-13R*α*2 chain) [47], human epidermal growth factor receptor 2 (HER2) [46], Ephrin type-A receptor 2 (EphA2) [48], gp100 [49], tenascin [50], survivin [51], melanoma antigen (MAGE)-1 [52], MAGE-3 [49], chitinase 3-like 1 (CHI3L1) [53], Wilms Tumor 1 Protein (WT-1) [54], SRY-related HMG-box gene (SOX)-11 and cytomegalovirus (CMV) antigens [55]. These have been over-expressed in GBMs and could initiate immune responses [56]. GAAs should be selected according to the human leukocyte antigen (HLA) genotype in each patient for HLA restriction in GBM [3]. However, GAAs often react weakly due to coexpression on normal tissues and subsequent host immunotolerance [55].

For GSAs, epidermal growth factor receptor variant III (EGFRvIII) is the only GSA targeted for GBM vaccination. It is present as a tumor-specific cell surface protein in 30–40% of GBM patients [57], and is absent on normal tissues, enhancing tumorigenicity [55]. In GBMs expressing EGFRvIII, DC vaccination can improve median progression-free survival (PFS) and median overall survival (OS) with minimal toxicity. When an anti-EGFRvIII DC vaccine was added to the standard therapy, patients had an increased PFS from 6.3 months to 14.2 months and improved OS from 15 to 26 months. Some vaccinated patients had serologic evidence of an anti-EGFRvIII humoral response to compete EGFRvIII-expressing GBM cells, and the median OS was 47.7 months in these patients compared to 22.8 months for vaccinated patients who did not develop serologic evidence of a humoral response. For patients who developed recurrent GBM after vaccination, pathological tissue demonstrated that recurrent GBM had lost EGFRvIII expression [58].

One patient newly diagnosed with GBM had obvious cytokine changes (related to DC vaccination) in IL-6, TNF-*α*, and IL-10 after receiving a GBM lysate-pulsed DC vaccination. Although cytokines declined after the first vaccine dose, IL-6 remained undetectable after all three doses, suggesting a potential antitumor immunological response [59].

CMV can modulate the malignant phenotype in GBMs, therefore DC vaccines pulsed with CMV antigens can be used to reduce GBM malignancy. After receiving a CMV peptide-pulsed DC vaccination, MS has been prolonged to 21 months in GBM patients [60].

Different GBM antigens offer varied efficacy of DC-based vaccination. Research to compare therapeutic efficacy of GAA peptide-loaded and autologous tumor lysate (ATL)-loaded DC vaccination in malignant glioma patients (most GBM patients) indicated that ATL-DC vaccination had greater feasibility for treatment and decreased fractions of activated natural killer (NK) cell populations (which were associated with prolonged survival in this trial), compared to GAA-DC vaccination [61].

Meta-analyses indicate that vaccination with whole-tumor antigens induced greater clinical responses than vaccination with defined tumor antigens for GAA expression heterogeneity [62, 63]. Single peptide vaccines can result in poor identification of specific GBM antigens for the escape of peptide-deficient variants; in consideration of the heterogeneous properties of GBM cells [64], most clinical trials with DC vaccination for GBM use whole-tumor lysates as sources of GAAs instead of artificially-synthesized peptides [3]. Genetic modification of DCs for antigen loading may also be an appropriate strategy, allowing multi-epitope presentation of full-length GAAs without HLA restrictions [43].

Whole GBM lysates can be generated from irradiation (apoptosis) or freeze-thawing (necrosis) of GBM cells. Lysates from apoptotic bodies increased the immunogenicity of GBM cells and enhanced GAA delivery to DCs more effectively than necrosis lysates [24]. However, loading DCs with apoptotic bodies of GBMs can increase risks in induction of tolerogenic DCs via the cyclooxygenase-2 (COX2) pathway [65]. DCs loaded with purified autophagosomes from autophagic tumor cells induced tumor-specific immune responses [66], and autophagy regulated selective release of high-mobility group B1 (HMGB1), which acted as an endogenous pattern recognition receptor (PRR) to induce DC maturation [67]. Therefore, autophagic tumor lysates and autophagosomes may be prudent choices for DC vaccines [66].

### 3.2. Glioma Stem Cell (GSC) Antigens

GSCs, a subpopulation that makes up 10–70% of the total cell population with GBMs, are closely related to GBM occurrence, progression, metastasis, recurrence, drug resistance and immune evasion [55, 68]. They possess higher immunogenicity compared with other tumor cells and can drive stronger immune responses [69]. Nestin (a type VI intermediate filament protein), CD15 and CD133, which exhibit different levels of expression in GBM cells, can be used as cell surface markers to isolate and characterize GSCs [70]. Nestin is broadly expressed in cancer stem cells (CSCs) from various malignancies, such as bladder, head and neck, ovarian, pancreatic, prostate, testicular, and uterine cancers [71–75]. CD15 is extensively expressed in thyroid, colorectal, lung, gastric, liver, nasopharynx, bladder cancer cells, while CD133 is broadly expressed in liver, lung, prostate, cerebral, colon, melanoma cancer cells as conventional CSC antigens [76].

GSCs can secrete several immunosuppressive cytokines associated with recruitment and polarization of microglia/macrophages, and they include: soluble colony-stimulating factor-1 (sCSF-1), transforming growth factor-*β*1 (TGF-*β*1) and macrophage inhibitory cytokine-1 (MIC-1). Moreover, conditioned media from GSCs polarize microglia/macrophages to an M2 phenotype, inhibit phagocytosis of microglia/macrophages, induce secretion of immunosuppressive cytokines (IL-10 and TGF-*β*1), and inhibit T cell proliferation [77, 78].

The resistance of GBM to radiotherapy and chemotherapy may be mediated by GSCs which have more active DNA repair mechanisms [79] and highly express multi-drug resistance genes [80], can be enriched via neurosphere culture conditions and contribute to local immunosuppression in the GBM microenvironment [9, 55, 68, 77]. Vaccine studies using lysates from CSCs revealed that superior protective immunity compared to lysates from whole tumors in mice [81, 82]. Therefore, GSCs antigens may be ideal for vaccination whether such a choice is more effective than other antigens [9, 55], and DCs loaded with GSC antigens may stimulate T cells to produce tumor-specific cytotoxicity against GBM cells [83].

Several vaccination in rodent orthotopic GBM models with DC loaded with GSC antigens, have been reported to induce immune-reactivity and a survival benefit [69, 81]. In DCs stimulated with lysates from GSCs, expression of DC surface molecules (including CD80, CD86, CD11C and MHC II) is upregulated more compared with DCs loaded with normal antigens and these more effectively stimulate naive T cells to form tumor-specific cytotoxic T cells that kill glioma cells cultured* in vitro* [83]. Thus, GSCs contain unknown antigens with strong immunogenicity that can be recognized by DCs, which need more researches (Table 3).

## 4. Regulation of the GBM Microenvironment

GBM cell immunogenicity depends on the microenvironment in which the cells grow. Many cytokines and other cells have unique roles in the GBM microenvironment, and some cause immune suppression. Thus, DC vaccination not only requires reduction of tumor load (tumor resection) as much as possible, but also calls for the regulation of the GBM microenvironment which including the addition of some positive regulators and the elimination of GBM-induced immune suppression [84] (Figure 1).

### 4.1. Positive Regulators

There are some positive regulators in the GBM microenvironment. The presence of some lymphocytes (such as CD8^+^ T cells [85], CD4^+^ T cells [86], and NK cells [87]), cytokines (such as type I IFN (IFN-*α* and IFN-*β*), and IL-12p70) in Th1-polarized microenvironments, can prime and activate antitumor cytotoxic and memory T cell responses [3].

Adoptively transferred tumor-specific T cells-especially those expressing chimeric antigen receptors (CARs)-enhanced immunity in preclinical studies, targeting several GBM antigens including: EGFRvIII, IL-13Ra2 chain, HER2, and CMV antigens [55, 88]. Research to study such efficacy by adding these cells to DC vaccines is forthcoming.

DCs can secrete cytokines and chemokines vital for immune polarization and recruitment of lymphocyte populations. Cytokine treatment is a powerful tool to induce robust anti-GBM cytotoxic and memory T-cell responses post-vaccination. IL-12p70 derived from DCs can stimulate IFN-*γ* production in naive T cells, promoting Th1 responses that overcome immune tolerance against tumor cells [3]. When Th1 cytokines such as IL-2 were pulsed to Flt3L-mediated gene therapy in a refractory rat model, therapeutic efficacy was strengthened by augmenting cytotoxic T lymphocyte responses and CD8^+^ T cell mediated immunological memory [89]. Increased responsiveness of CD8^+^ T lymphocytes to IL-2 was related to long-term survival of greater than 2 years post-vaccination in GBM patients. This technique activated the JAK-STAT signal pathways, causing phosphorylation of STAT-5 via cytokine receptors located on the T cell membrane [90]. Dimers then formed from the phosphorylated STATs (pSTAT) and trans-located into the nucleus to initiate gene transcription programs. Perhaps GBM induces immune suppression against IL-2 signals in T cells, and then DC vaccines counteract immune suppression [91]. STAT-5 is required for IL-2-induced cell cycle progression in T cells and the recruitment of antibody-induced T cells into tumor tissues [92]. Enhanced sensitivity to IL-2 signals and increased frequency of pSTAT-5 upregulate the clinical responsiveness of IL-2-primed CD8^+^ T cells to intracranial tumors [93], and STAT5-deficient mice had altered NK cell function and decreased T and B cell proliferation in response to chemokines [94]. Adding recombinant IL-12 to lysates or RNA loaded DCs also can strengthen protective immunity against intracranial gliomas [95] and combining DC vaccination with IFN-*β* gene therapy benefits survival [96]. Long-term survival and specific cytotoxic T lymphocyte activity was induced when IFN-*α* was delivered in sequential pulses to DC vaccines in a mouse glioma model [97]. Thus, local proinflammatory cytokine production post-vaccination can affect the generation of effector memory CD8^+^ T lymphocyte populations [91], thereby ulteriorly influence the efficacy of DC vaccination. Since circulating cytokines demonstrate little relation to intracranial immune responses [59], intratumoral injection of immunostimulatory cytokines can prolong the survival of DCs administered subcutaneously.

### 4.2. Negative Regulators

Inhibitory cytokines as well as suppressive cell populations secreted by GBM cells and the existence of vascular endothelial growth factor (VEGF) in the GBM microenvironment are negative regulators in the immunity targeting GBMs.

Inhibitory cytokines such as transforming growth factor (TGF)-*β* in the GBM microenvironment prevent the immune response from translating into clinical efficacy [98, 99]. Substances that interfere with the TGF–*β* signal pathways have been tested in early clinical trials including inactivating antibodies (fresolimumab) and antisense oligonucleotides (trabedersen) [64, 100, 101], which may provide a solution to the infiltration of TGF-*β* and enhance antitumor immunity of DC-based vaccination.

Immune regulatory components such as Tregs or myeloid-derived suppressor cells (MDSCs) in the GBM microenvironmentcan cause immune tolerance. Tregs are a subpopulation of CD4^+^ T lymphocytes [102]. Infiltration with Tregs is associated with glioma progression [103] and inhibiting CD4^+^ T cells, CD8^+^ T cells, DCs, and NK cells hinders a successful immune response [104–106]. Decreased post/pre-vaccination Tregs ratios were reported to be related to prolonged survival in glioma patients [61], so preventing or reversing these components through inhibition can enhance antitumor immunity of DC-based vaccination [3]. Depleting Tregs via antibody treatment to modulate the tumor microenvironment [99] can permit the generation of effective antitumor responses [104, 105] and these substances are attractive when combining with DC vaccination [64]. Tregs constitutively express the high affinity IL-2 receptor CD25, the transcription factor Forkhead box protein 3 (Foxp3) and the B7 ligand CTLA4 [102], all of which can be target for the depletion of Tregs. Immune responses were significantly enhanced after DC vaccination in GBM patients who received CD25 mAb blockade (daclizumab) and temozolomide chemotherapy [107]. However, CD25 is not a specific marker for Tregs. In a large glioma model, the depletion of Tregs by using blockade of CD25 strategy inhibited the clonal expansion of tumor specific T cells and decreased the efficacy of DC vaccines [108].

Foxp3 is a more specific marker expressed by Tregs in human GBM as compared to CD25 and it may be a target for Treg depletion. Foxp3 also suppresses IFN-*γ* and IL-2 secretion from CD4^+^ T cells [109]. However, Foxp3 is intranuclear and cannot be depleted easily with immunoglobulins [110]. Delivery of inhibitors to NF-кB combined with immunogene therapy using Flt3L and thymidine kinase (TK) can suppress Foxp3^+^ Tregs and product Th1 cytokines in the tumor microenvironment [89]. Another option exists for eliminating Tregs is anticytotoxic T-lymphocyte antigen 4 (CTLA4) antibodies. Ipilimumab, a monoclonal antibody targeting CTLA4, will soon be approved by FDA for advanced melanoma [32], including those with CNS metastases. This may be feasible for use in immunotherapy for GBM [55]. One study suggested that Treg depletion (using a CD25-targetting strategy that interfered with the clonal expansion of tumor antigen specific T lymphocytes) inhibited the efficiency of DC-based immunotherapy in a glioma model [108]. Therefore, the efficacy of Tregs depletion in GBM needs further researches.

MDSCs are also negative regulators for antitumor immunity. Although coculture of normal human monocytes with glioma cells* in vitro* acquired MDSC-like properties [111], GBM patients had increased MDSCs (CD33^+^ HLA-DR^−^) in peripheral blood compared to normal donors. MDSCs isolated from peripheral blood monocytes (PBMs) significantly restored T-cell function [112]. Thus, MDSCs are related to GBM tolerance and can be combined with DC vaccines. COX-2 inhibition (celecoxib) or anti-Gri antibody can block the development of MDSCs (CD11b^+^ Gri^+^) as well as CCL2-mediated accumulation in the GBM microenvironment and delay glioma development in a murine model [113, 114]. Moreover, accumulating evidence suggests that several chemotherapeutic agents (gemcitabine, docetaxel, 5-fluorouracil, and sunitinib malate, a receptor tyrosine kinase inhibitor) could reverse immune suppression mediated by MDSCs in mouse tumor models. Other compounds such as polyphenol E or all-trans-retinoic acid also can decrease MDSCs in mice and humans [3].

Vascular endothelial growth factor (VEGF) is another negative regulator which contributes to the immunosuppressive ability of GBMs. It can inhibit the maturation of DCs and antigen presentation, induce apoptosis of CD8^+^ T cells, enhance Treg activity and diminish the infiltration of T cells in GBM endothelium. The inhibition of VEGF can block VEGF mediated angiogenesis of GBM and also suppress the growth of GSC-derived tumor cells [115]. Bevacizumab, a humanized monoclonal antibody which blocks VEGF mediated angiogenesis of GBM. This is an approved therapy for recurrent GBM by FDA and has been shown to be efficacious in newly diagnosed GBM patients in phase III clinical trials [55]. VEGF Trap such as aflibercept is also one of VEGF-targeting drugs. It has greater affinity for VEGF compared to anti-VEGF monoclonal antibodies and has improved survival as well as enhanced the activity of radiation therapy in preclinical studies. Meanwhile, GBM expresses VEGF receptors (VEGFRs) which may promote tumor growth. Therapies target VEGFRs by suppressing activation signaling of VEGFR can also effect. The activation of VEGFRs can be inhibited by blocking the tyrosine kinase activation site of VEGFR with tyrosine kinase inhibitors or blocking the ligand binding site of VEGFR with monoclonal antibodies or peptides. Several VEGFR tyrosine kinase inhibitors such as cediranib have induced powerful antiangiogenic and antitumor activity in preclinical GBM models. Considering these molecules also inhibit other relevant receptors, they may also increase toxicity [115] (Table 4).

## 5. DC-Based Vaccination 

Once extracted from humans, DCs can be exposed to antigens expressed by GBM cells, and stimulated to take up, process, and display these antigens as peptides on their cell surface in the context of MHC class I or II molecules. These cells can then be infused back into patients as a vaccine therapy. T cells can be activated by recognizing MHC class I or II molecules via TCRs. Vaccines also induce cross-stimulation of CD8^+^ (cytotoxic) T-cell (CTL) responses, as well as Th1 and Th2 pathways by stimulating differentiation of naïve CD4^+^ T cells into helper T effectors [116, 117], which may be more effective than stimulating immunity using MHC I restricted peptides only [59]. Stimulated CD8^+^ CTLs secrete IFN-*γ* and have potent cytolytic activity against GBM cells now recognized by the host's immune system [3, 9], whereby they recognize and destroy GBM cells via peptides derived from GAAs of MHC class I molecules. Meanwhile, activated CD4^+^ T cells recognize peptides in the complex of MHC class II molecules and improve the capacity of DCs to induce CTLs via interaction between CD40 ligands on activated CD4^+^ T cells and CD40 on DCs. Moreover, CD4^+^ T cells maintain and expand CTLs by secreting cytokines such as IL-12. Not only can DCs elicit T-cell responses, but also they can improve the immunomodulatory and cytotoxic potential of NK and natural killer T (NKT) cells [5], both of which are also involved in the elimination of GBM. Furthermore, they also mediate tumor-directed cytotoxicity directly [9, 56] (Figure 1). In brief, DC vaccines can active patients' immune systems and strengthen the immune responses against GBM cells.

## 6. Summary

As a basic immunotherapy, DC vaccination is critical for initiating and boosting anti-GBM immunity; it has obvious complementarity with traditional treatments in promoting cross-presentation of antigens and long term immunologic memory, and it can prevent the recurrence and metastases of GBM [10]. Therefore, the combination of DC-based vaccination with traditional modalities may offer promise for novel GBM treatments [3]. Besides, the antitumor immunity of DC vaccination can be strengthened by inhibiting inhibitory signals or components; upregulating stimulatory molecules or signals; choosing specific antigens for loading; and regulating the GBM microenvironment. However, DC vaccines have had several limitations so far which include a surgical requirement, a several-week delay for vaccine generation, the possibility of immune overload due to excessive antigen exposure, and potential autoimmune reactions which due to vaccine contamination with normal host [55].

## Figures and Tables

**Algorithm 1:**
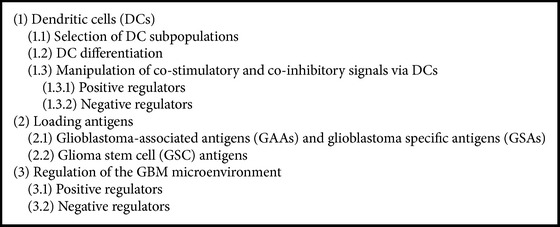
Classification scheme of approaches to strengthen the efficacy of DC vaccines in the treatment of glioblastoma.

**Table 1:** Comparison between GM-CSF and Flt3L in culturing DCs. GM-CSF: granulocyte macrophage colony-stimulating factor, Flt3L: FMS-like tyrosine kinase-3 ligand, DC: dendritic cell, DC1: type 1 polarizing DC, DC2: type 2 polarizing DC, Th1: helper T cell 1, and Th2: helper T cell 2.

	GM-CSF	Flt3L
DC subsets expending [17]	DC1 < DC2	DC1 > DC2
T cells expanding [17]		More memory CD4^+^, CD8^+^ T cells
Helper T cells expanding [17]	Both Th1 and Th2	Mainly Th1

**Table 2:** Positive and negative regulators on DCs. TNF: tumor necrosis factor, ICOS: inducible costimulator, TNFRSF4: tumor necrosis factor receptor superfamily-member 4, TNFSF14: tumor necrosis factor ligand superfamily-member 14, GITR: glucocorticoid-induced tumor necrosis factor receptor, PD-L1: programmed death 1 ligand, A20: zinc finger protein A20, SOCS1: the suppressor of cytokine signaling 1, and DIgR2: DC-derived immunoglobulin receptor 2.

Positive regulators		Negative regulators
B7/CD28 family	TNF/TNF receptor family	
CD80 (B7-1) [25] CD86 (B7-2) [25] B7-H2 (ICOS ligand) [26] B7-H3 [27]	CD27 [30] TNFRSF4 [31] CD137 (4-1 BB) [30] TNFSF14 [29] GITR [30]	PD-L1 (B7-H1) [22] PD-L2 (B7-DC) [40] B7-H4 [28] A20 [42] SOCS1 [43] DIgR2 [44] Notch ligands [44]

**Table 3:** Antigens for loading on DCs. AIM-2: antigen isolated from immunoselected melanoma-2, IL-13R*α*2 chain: the *α*-2 chain of the IL-13 receptor, HER2: human epidermal growth factor receptor 2, EphA2: Ephrin type-A receptor 2, MAGE: melanoma antigen, CHI3L1: chitinase 3-like 1, WT-1: Wilms Tumor 1 Protein, SOX: SRY-related HMG-box gene, CMV: cytomegalovirus, EGFR: epidermal growth factor receptor, and EGFRvIII: epidermal growth factor receptor variant III.

GAAs	GSAs	GSC antigens
AIM-2 [46], IL-13R*α*2 chain [47], HER2 [46], EphA2 [48], gp100 [49], tenascin [50], surviving [51], MAGE-1 [52], MAGE-3 [49], CHI3L1 [53], WT-1 [54], SOX11 [55], CMV antigens [55]	EGFRvIII [57]	EGFR [81], SOX2 [81]

**Table 4:** Regulators in the GBM microenvironment. GBM: glioblastoma multiforme, NK: natural killer; IFN: interferon; IL: interleukin, Tregs: regulatory T cells, MDSCs: myeloid-derived suppressor cells, TGF: transforming growth factor, and VEGF: vascular endothelial growth factor.

Positive regulators		Negative regulators	
Cells	Cytokines	Cells	Cytokines
CD8^+^ T cells [85] CD4^+^ T cells [86] NK cells [87]	IFN-*α* [97] IFN-*β* [96] IL-12p70 [3] IL-2 [89] IL-12 [95]	Tregs [103] MDSCs [112]	TGF-*β* [98] VEGF [115]

## References

[1] Wen P. Y., Kesari S. (2008). Malignant gliomas in adults. *New England Journal of Medicine*.

[2] Jovcevska I., Kocevar N., Komel R. (2013). Glioma and glioblastoma—how much do we (not) know?. *Molecular Clinical Oncology*.

[3] Mineharu Y., Castro M. G., Lowenstein P. R., Sakai N., Miyamoto S. (2013). Dendritic Cell-based Immunotherapy for glioma: multiple regimens and implications in clinical trials. *Neurologia Medico-Chirurgica*.

[4] Gruber M. L., Buster W. P. (2004). Temozolomide in combination with irinotecan for treatment of recurrent malignant glioma. *American Journal of Clinical Oncology*.

[5] Yang M.-Y., Zetler P. M., Prins R. M., Khan-Farooqi H., Liau L. M. (2006). Immunotherapy for patients with malignant glioma: from theoretical principles to clinical applications. *Expert Review of Neurotherapeutics*.

[6] Cheever M. A., Higano C. S. (2011). PROVENGE (Sipuleucel-T) in prostate cancer: the first FDA-approved therapeutic cancer vaccine. *Clinical Cancer Research*.

[7] Traynor K. (2011). Ipilimumab approved for metastatic melanoma. *The American Journal of Health-System Pharmacy*.

[8] Bregy A., Wong T. M., Shah A. H., Goldberg J. M., Komotar R. J. (2013). Active immunotherapy using dendritic cells in the treatment of glioblastoma multiforme. *Cancer Treatment Reviews*.

[9] Marsh J. C., Goldfarb J., Shafman T. D., Diaz A. Z. (2013). Current status of immunotherapy and gene therapy for high-grade gliomas. *Cancer Control*.

[10] Butterfield L. H. (2013). Dendritic cells in cancer immunotherapy clinical trials: are we making progress?. *Frontiers in Immunology*.

[11] Trombetta E. S., Mellman I. (2005). Cell biology of antigen processing in vitro and in vivo. *Annual Review of Immunology*.

[12] Cohn L., Delamarre L. (2014). Dendritic cell-targeted vaccines. *Frontiers in Immunology*.

[13] Jie X., Hua L., Jiang W., Feng F., Feng G., Hua Z. (2012). Clinical application of a dendritic cell vaccine raised against heat-shocked glioblastoma. *Cell Biochemistry and Biophysics*.

[14] Napolitani G., Rinaldi A., Bertoni F., Sallusto F., Lanzavecchia A. (2005). Selected Toll-like receptor agonist combinations synergistically trigger a T helper type 1-polarizing program in dendritic cells. *Nature Immunology*.

[15] Fujita M., Zhu X., Ueda R. (2009). Effective immunotherapy against murine gliomas using type 1 polarizing dendritic cells—significant roles of CXCL10. *Cancer Research*.

[16] Mailliard R. B., Wankowicz-Kalinska A., Cai Q. (2004). Alpha-type-1 polarized dendritic cells: a novel immunization tool with optimized CTL-inducing activity. *Cancer Research*.

[17] Parajuli P., Mosley R. L., Pisarev V. (2001). Flt3 ligand and granulocyte-macrophage colony-stimulating factor preferentially expand and stimulate different dendritic and T-cell subsets. *Experimental Hematology*.

[18] Weigel B. J., Nath N., Taylor P. A. (2002). Comparative analysis of murine marrow-derived dendritic cells generated by Flt3L or GM-CSF/IL-4 and matured with immune stimulatory agents on the in vivo induction of antileukemia responses. *Blood*.

[19] Cohen P. A., Koski G. K., Czerniecki B. J. (2008). STAT3-And STAT5-dependent pathways competitively regulate the pan-differentiation of CD34 pos cells into tumor-competent dendritic cells. *Blood*.

[20] Berhanu A., Huang J., Alber S. M., Watkins S. C., Storkus W. J. (2006). Combinational FLt3 ligand and granulocyte macrophage colony-stimulating factor treatment promotes enhanced tumor infiltration by dendritic cells and antitumor CD8^+^ T-cell cross-priming but is ineffective as a therapy. *Cancer Research*.

[21] Iellem A., Mariani M., Lang R. (2001). Unique chemotactic response profile and specific expression of chemokine receptors CCR4 and CCR8 by CD4(+)CD25(+) regulatory T cells. *The Journal of Experimental Medicine*.

[22] Capece D., Verzella D., Fischietti M., Zazzeroni F., Alesse E. (2012). Targeting costimulatory molecules to improve antitumor immunity. *Journal of Biomedicine and Biotechnology*.

[23] Lafferty K. J., Warren H. S., Woolnough J. A. (1979). A mediator acting as a costimulator for the development of cytotoxic responses in vitro. *Advances in Experimental Medicine and Biology*.

[24] Mineharu Y., King G. D., Muhammad A. G. (2011). Engineering the brain tumor microenvironment enhances the efficacy of dendritic cell vaccination: implications for clinical trial design. *Clinical Cancer Research*.

[25] Driessens G., Kline J., Gajewski T. F. (2009). Costimulatory and coinhibitory receptors in anti-tumor immunity. *Immunological Reviews*.

[26] Strauss L., Bergmann C., Szczepanski M. J., Lang S., Kirkwood J. M., Whiteside T. L. (2008). Expression of ICOS on human melanoma-infiltrating CD4^+^CD25 ^high^Foxp3^+^ T regulatory cells: implications and impact on tumor-mediated immune suppression. *Journal of Immunology*.

[27] Greenwald R. J., Freeman G. J., Sharpe A. H. (2005). The B7 family revisited. *Annual Review of Immunology*.

[28] Sica G. L., Choi I. H., Zhu G. (2003). B7-H4, a molecule of the B7 family, negatively regulates T cell immunity. *Immunity*.

[29] del Rio M. L., Lucas C. L., Buhler L., Rayat G., Rodriguez-Barbosa J. I. (2010). HVEM/LIGHT/BTLA/CD160 cosignaling pathways as targets for immune regulation. *Journal of Leukocyte Biology*.

[30] Watts T. H. (2005). TNF/TNFR family members in costimulation of T cell responses. *Annual Review of Immunology*.

[31] Croft M. (2003). Costimulation of T cells by OX40, 4-1BB, and CD27. *Cytokine and Growth Factor Reviews*.

[32] Hodi F. S., O'Day S. J., McDermott D. F. (2010). Improved survival with ipilimumab in patients with metastatic melanoma. *The New England Journal of Medicine*.

[33] Bonifaz L., Bonnyay D., Mahnke K., Rivera M., Nussenzweig M. C., Steinman R. M. (2002). Efficient targeting of protein antigen to the dendritic cell receptor DEC-205 in the steady state leads to antigen presentation on major histocompatibility complex class I products and peripheral CD8^+^ T cell tolerance. *The Journal of Experimental Medicine*.

[34] Lim S. N., Kuhn S., Hyde E., Ronchese F. (2012). Combined TLR stimulation with Pam3Cys and Poly I: C enhances Flt3-ligand dendritic cell activation for tumor immunotherapy. *Journal of Immunotherapy*.

[35] Prins R. M., Craft N., Bruhn K. W. (2006). The TLR-7 agonist, imiquimod, enhances dendritic cell survival and promotes tumor antigen-specific T cell priming: Relation to central nervous system antitumor immunity. *Journal of Immunology*.

[36] Grauer O. M., Molling J. W., Bennink E. (2008). TLR ligands in the local treatment of established intracerebral murine gliomas. *Journal of Immunology*.

[37] Stone G. W., Barzee S., Snarsky V. (2009). Nanoparticle-delivered multimeric soluble CD40L DNA combined with toll-like receptor agonists as a treatment for melanoma. *PLoS ONE*.

[38] Calabi F., Jarvis J. M., Martin L., Milstein C. (1989). Two classes of CD1 genes. *European Journal of Immunology*.

[39] Hunn M. K., Hermans I. F. (2013). Exploiting invariant NKT cells to promote T-cell responses to cancer vaccines. *OncoImmunology*.

[40] Latchman Y., Wood C. R., Chernova T. (2001). PD-L2 is a second ligand for PD-1 and inhibits T cell activation. *Nature Immunology*.

[41] Brown J. A., Dorfman D. M., Ma F.-R. (2003). Blockade of programmed death-1 ligands on dendritic cells enhances T cell activation and cytokine production. *The Journal of Immunology*.

[42] Boone D. L., Turer E. E., Lee E. G. (2004). The ubiquitin-modifying enzyme A20 is required for termination of Toll-like receptor responses. *Nature Immunology*.

[43] Boudreau J. E., Bonehill A., Thielemans K., Wan Y. (2011). Engineering dendritic cells to enhance cancer immunotherapy. *Molecular Therapy*.

[44] Stallwood Y., Briend E., Ray K. M. (2006). Small interfering RNA-mediated knockdown of notch ligands in primary CD4^+^ T cells and dendritic cells enhances cytokine production. *The Journal of Immunology*.

[45] Gu J.-H., Li G. (2008). Dendritic cell-based immunotherapy for malignant glioma. *Neuroscience Bulletin*.

[46] Phuphanich S., Wheeler C. J., Rudnick J. D. (2013). Phase i trial of a multi-epitope-pulsed dendritic cell vaccine for patients with newly diagnosed glioblastoma. *Cancer Immunology, Immunotherapy*.

[47] Okano F., Storkus W. J., Chambers W. H., Pollack I. F., Okada H. (2002). Identification of a novel HLA-A^*^0201-restricted, cytotoxic T lymphocyte epitope in a human glioma-associated antigen, interleukin 13 receptor *α*2 chain. *Clinical Cancer Research*.

[48] Hatano M., Eguchi J., Tatsumi T. (2005). EphA2 as a glioma-associated antigen: a novel target for glioma vaccines. *Neoplasia*.

[49] Saikali S., Avril T., Collet B. (2007). Expression of nine tumour antigens in a series of human glioblastoma multiforme: interest of EGFRvIII, IL-13Ralpha2, gp100 and TRP-2 for immunotherapy. *Journal of Neuro-Oncology*.

[50] Brösicke N., van Landeghem F. K. H., Scheffler B., Faissner A. (2013). Tenascin-C is expressed by human glioma in vivo and shows a strong association with tumor blood vessels. *Cell and Tissue Research*.

[51] Guo H., Wang Y., Song T. (2014). Silencing of survivin using YM155 inhibits invasion and suppresses proliferation in glioma cells. *Cell Biochemistry and Biophysics*.

[52] Liu G., Ying H., Zeng G., Wheeler C. J., Black K. L., Yu J. S. (2004). HER-2, gp100, and MAGE-1 are expressed in human glioblastoma and recognized by cytotoxic T cells. *Cancer Research*.

[53] Nutt C. L., Betensky R. A., Brower M. A., Batchelor T. T., Louis D. N., Stemmer-Rachamimov A. O. (2005). YKL-40 is a differential diagnostic marker for histologic subtypes of high-grade gliomas. *Clinical Cancer Research*.

[54] Chiba Y., Kinoshita M., Okita Y. (2012). Use of 11C-methionine PET parametric response map for monitoring WT1 immunotherapy response in recurrent malignant glioma. *Journal of Neurosurgery*.

[55] Reardon D. A., Wucherpfennig K. W., Freeman G. (2013). An update on vaccine therapy and other immunotherapeutic approaches for glioblastoma. *Expert Review of Vaccines*.

[56] Xu X., Stockhammer F., Schmitt M. (2012). Cellular-based immunotherapies for patients with glioblastoma multiforme. *Clinical and Developmental Immunology*.

[57] Wong A. J., Ruppert J. M., Bigner S. H. (1992). Structural alterations of the epidermal growth factor receptor gene in human gliomas. *Proceedings of the National Academy of Sciences of the United States of America*.

[58] Sampson J. H., Heimberger A. B., Archer G. E. (2010). Immunologic escape after prolonged progression-free survival with epidermal growth factor receptor variant III peptide vaccination in patients with newly diagnosed glioblastoma. *Journal of Clinical Oncology*.

[59] Lasky J. L., Panosyan E. H., Plant A. (2013). Autologous tumor lysate-pulsed dendritic cell immunotherapy for pediatric patients with newly diagnosed or recurrent high-grade gliomas. *Anticancer Research*.

[60] Dziurzynski K., Chang S. M., Heimberger A. B. (2012). Consensus on the role of human cytomegalovirus in glioblastoma. *Neuro-Oncology*.

[61] Prins R. M., Wang X., Soto H. (2013). Comparison of glioma-associated antigen peptide-loaded versus autologous tumor lysate-loaded dendritic cell vaccination in malignant glioma patients. *Journal of Immunotherapy*.

[62] Neller M. A., López J. A., Schmidt C. W. (2008). Antigens for cancer immunotherapy. *Seminars in Immunology*.

[63] Nava S., Dossena M., Pogliani S. (2012). An optimized method for manufacturing a clinical scale dendritic cell-based vaccine for the treatment of glioblastoma. *PLoS ONE*.

[64] Eyrich M., Rachor J., Schreiber S. C., Wolfl M., Schlegel P. G. (2013). Dendritic cell vaccination in pediatric Gliomas: lessons learnt and future perspectives. *Frontiers in Pediatrics*.

[65] Akasaki Y., Liu G., Chung N. H. C., Ehtesham M., Black K. L., Yu J. S. (2004). Induction of a CD4^+^ T regulatory type 1 response by cyclooxygenase-2-overexpressing glioma. *The Journal of Immunology*.

[66] Li Y., Wang L.-X., Yang G., Hao F., Urba W. J., Hu H.-M. (2008). Efficient cross-presentation depends on autophagy in tumor cells. *Cancer Research*.

[67] Thorburn J., Horita H., Redzic J., Hansen K., Frankel A. E., Thorburn A. (2009). Autophagy regulates selective HMGB1 release in tumor cells that are destined to die. *Cell Death and Differentiation*.

[68] Prestegarden L., Enger P. Ø. (2010). Cancer stem cells in the central nervous system—a critical review. *Cancer Research*.

[69] Pellegatta S., Poliani P. L., Corno D. (2006). Neurospheres enriched in cancer stem-like cells are highly effective in eliciting a dendritic cell-mediated immune response against malignant gliomas. *Cancer Research*.

[70] Jin X., Jung J.-E., Beck S., Kim H. (2013). Cell surface Nestin is a biomarker for glioma stem cells. *Biochemical and Biophysical Research Communications*.

[71] Singh S. K., Clarke I. D., Hide T., Dirks P. B. (2004). Cancer stem cells in nervous system tumors. *Oncogene*.

[72] Kasper S. (2008). Exploring the origins of the normal prostate and prostate cancer stem cell. *Stem Cell Reviews*.

[73] Bentivegna A., Conconi D., Panzeri E. (2010). Biological heterogeneity of putative bladder cancer stem-like cell populations from human bladder transitional cell carcinoma samples. *Cancer Science*.

[74] Okuno K., Ohta S., Kato H., Taga T., Sugita K., Takeuchi Y. (2010). Expression of neural stem cell markers in malignant rhabdoid tumor cell lines. *Oncology Reports*.

[75] Jimeno A., Feldmann G., Suárez-Gauthier A. (2009). A direct pancreatic cancer xenograft model as a platform for cancer stem cell therapeutic development. *Molecular Cancer Therapeutics*.

[76] Corbeil D., Röper K., Hellwig A. (2000). The human AC133 hematopoietic stem cell antigen is also expressed in epithelial cells and targeted to plasma membrane protrusions. *The Journal of Biological Chemistry*.

[77] Wu A., Wei J., Kong L.-Y. (2010). Glioma cancer stem cells induce immunosuppressive macrophages/microglia. *Neuro-Oncology*.

[78] di Tomaso T., Mazzoleni S., Wang E. (2010). Immunobiological characterization of cancer stem cells isolated from glioblastoma patients. *Clinical Cancer Research*.

[79] Bao S., Wu Q., McLendon R. E. (2006). Glioma stem cells promote radioresistance by preferential activation of the DNA damage response. *Nature*.

[80] Bleau A.-M., Hambardzumyan D., Ozawa T. (2009). PTEN/PI3K/Akt pathway regulates the side population phenotype and ABCG2 activity in glioma tumor stem-like cells. *Cell Stem Cell*.

[81] Xu Q., Liu G., Yuan X. (2009). Antigen-specific T-cell response from dendritic cell vaccination using cancer stem-like cell-associated antigens. *Stem Cells*.

[82] Ghods A. J., Irvin D., Liu G. (2007). Spheres isolated from 9L gliosarcoma rat cell line possess chemoresistant and aggressive cancer stem-like cells. *Stem Cells*.

[83] Ji B., Chen Q., Liu B. (2013). Glioma stem cell-targeted dendritic cells as a tumor vaccine against malignant glioma. *Yonsei Medical Journal*.

[84] Hatiboglu M. A., Wei J., Wu A. S. G., Heimberger A. B. (2010). Immune therapeutic targeting of glioma cancer stem cells. *Targeted Oncology*.

[85] Kalinski P., Nakamura Y., Watchmaker P., Giermasz A., Muthuswamy R., Mailliard R. B. (2006). Helper roles of NK and CD8^+^ T cells in the induction of tumor immunity. Polarized dendritic cells as cancer vaccines. *Immunologic Research*.

[86] Koido S., Enomoto Y., Apostolopoulos V., Gong J. (2014). Tumor regression by CD4 T-cells primed with dendritic/tumor fusion cell vaccines. *Anticancer Research*.

[87] Wargo J. A., Schumacher L. Y., Comin-Anduix B. (2005). Natural killer cells play a critical role in the immune response following immunization with melanoma-antigen-engineered dendritic cells. *Cancer Gene Therapy*.

[88] Prins R. M., Shu C. J., Radu C. G. (2008). Anti-tumor activity and trafficking of self, tumor-specific T cells against tumors located in the brain. *Cancer Immunology, Immunotherapy*.

[89] Mineharu Y., Muhammad A. G., Yagiz K. (2012). Gene therapy-mediated reprogramming tumor infiltrating T cells using IL-2 and inhibiting NF-kappaB signaling improves the efficacy of immunotherapy in a brain cancer model. *Neurotherapeutics*.

[90] Ihle J. N., Kerr I. M. (1995). Jaks and Stats in signaling by the cytokine receptor superfamily. *Trends in Genetics*.

[91] Everson R. G., Jin R. M., Wang X. (2014). Cytokine responsiveness of CD8^+^ T cells is a reproducible biomarker for the clinical efficacy of dendritic cell vaccination in glioblastoma patients. *Journal for ImmunoTherapy of Cancer*.

[92] Moriggl R., Topham D. J., Teglund S. (1999). Stat5 is required for IL-2-induced cell cycle progression of peripheral T cells. *Immunity*.

[93] Lisiero D. N., Soto H., Liau L. M., Prins R. M. (2011). Enhanced sensitivity to IL-2 signaling regulates the clinical responsiveness of IL-12-primed CD8^+^ T cells in a melanoma model. *Journal of Immunology*.

[94] Akira S. (1999). Functional roles of STAT family proteins: lessons from knockout mice. *Stem Cells*.

[95] Insug O., Ku G., Ertl H. C. J., Blaszczyk-Thurin M. (2002). A dendritic cell vaccine induces protective immunity to intracranial growth of glioma. *Anticancer Research*.

[96] Saito R., Mizuno M., Nakahara N. (2004). Vaccination with tumor cell lysate-pulsed dendritic cells augments the effect of *IFN-β* gene therapy for malignant glioma in an experimental mouse intracranial glioma. *International Journal of Cancer*.

[97] Okada H., Kalinski P., Ueda R. (2011). Induction of CD8^+^ T-cell responses against novel glioma-associated antigen peptides and clinical activity by vaccinations with *α*-type 1 polarized dendritic cells and polyinosinic-polycytidylic acid stabilized by lysine and carboxymethylcellulose in patients with recurrent malignant glioma. *Journal of Clinical Oncology*.

[98] Siepl C., Bodmer S., Frei K. (1988). The glioblastoma-derived T cell suppressor factor/transforming growth factor-*β*2 inhibits T cell growth without affecting the interaction of interleukin 2 with its receptor. *European Journal of Immunology*.

[99] Maes W., Rosas G. G., Verbinnen B. (2009). DC vaccination with anti-CD25 treatment leads to long-term immunity against experimental glioma. *Neuro-Oncology*.

[100] Trachtman H., Fervenza F. C., Gipson D. S. (2011). A phase 1, single-dose study of fresolimumab, an anti-TGF-*Β* antibody, in treatment-resistant primary focal segmental glomerulosclerosis. *Kidney International*.

[101] Bogdahn U., Hau P., Stockhammer G. (2011). Targeted therapy for high-grade glioma with the TGF-*β*2 inhibitor trabedersen: results of a randomized and controlled phase IIb study. *Neuro-Oncology*.

[102] Qu Y., Zhao Y. (2007). Regulatory CD4^+^CD25^+^ T-cells are controlled by multiple pathways at multiple levels. *International Reviews of Immunology*.

[103] Grauer O. M., Nierkens S., Bennink E. (2007). CD4+FoxP3+ regulatory T cells gradually accumulate in gliomas during tumor growth and efficiently suppress antiglionia immune responses in vivo. *International Journal of Cancer*.

[104] Grauer O. M., Sutmuller R. P. M., van Maren W. (2008). Elimination of regulatory T cells is essential for an effective vaccination with tumor lysate-pulsed dendritic cells in a murine glioma model. *International Journal of Cancer*.

[105] Morse M. A., Hobeika A. C., Osada T. (2008). Depletion of human regulatory T cells specifically enhances antigen-specific immune responses to cancer vaccines. *Blood*.

[106] Thomas A. A., Ernstoff M. S., Fadul C. E. (2012). Immunotherapy for the treatment of glioblastoma. *Cancer Journal*.

[107] Mitchell D. A., Cui X., Schmittling R. J. (2011). Monoclonal antibody blockade of IL-2 receptor *α* during lymphopenia selectively depletes regulatory T cells in mice and humans. *Blood*.

[108] Curtin J. F., Candolfi M., Fakhouri T. M. (2008). Treg depletion inhibits efficacy of cancer immunotherapy: implications for clinical trials. *PLoS ONE*.

[109] Bettelli E., Dastrange M., Oukka M. (2005). Foxp3 interacts with nuclear factor of activated T cells and NF-kappaB to repress cytokine gene expression and effector functions of T helper cells. *Proceedings of the National Academy of Sciences of the United States of America*.

[110] Park J. H., Ko J. S., Shin Y. (2014). Intranuclear interactomic inhibition of FoxP3 suppresses functions of Treg cells. *Biochemical and Biophysical Research Communications*.

[111] Rodrigues J. C., Gonzalez G. C., Zhang L. (2010). Normal human monocytes exposed to glioma cells acquire myeloid-derived suppressor cell-like properties. *Neuro-Oncology*.

[112] Raychaudhuri B., Ireland P. R. J., Ko J. (2011). Myeloid-derived suppressor cell accumulation and function in patients with newly diagnosed glioblastoma. *Neuro-Oncology*.

[113] Zhang H., Tian M., Xiu C., Wang Y., Tang G. (2013). Enhancement of antitumor activity by combination of tumor lysate-pulsed dendritic cells and celecoxib in a rat glioma model. *Oncology Research*.

[114] Fujita M., Kohanbash G., Fellows-Mayle W. (2011). COX-2 blockade suppresses gliomagenesis by inhibiting myeloid-derived suppressor cells. *Cancer Research*.

[115] Reardon D. A., Turner S., Peters K. B. (2011). A review of VEGF/VEGFR-targeted therapeutics for recurrent glioblastoma. *Journal of the National Comprehensive Cancer Network*.

[116] Kim W., Liau L. M. (2010). Dendritic cell vaccines for brain tumors. *Neurosurgery Clinics of North America*.

[117] Knutson K. L., Disis M. L. (2005). Tumor antigen-specific T helper cells in cancer immunity and immunotherapy. *Cancer Immunology, Immunotherapy*.

